# Reduced Time to Breast Cancer Diagnosis with Coordination of Radiological and Clinical Care

**DOI:** 10.7759/cureus.1919

**Published:** 2017-12-07

**Authors:** Elaine C McKevitt, Carol K Dingee, Sher-Ping Leung, Carl J Brown, Nancy Y Van Laeken, Richard Lee, Urve Kuusk

**Affiliations:** 1 Surgery, Providence Health Care; 2 Mt. St Joseph Hospital, University of British Columbia Vancouver; 3 Surgery, Chilliwack General Hospital; 4 Surgery, University of British Columbia Vancouver; 5 Plastic Surgery, Providence Health Care; 6 Radiology, Msj Hospital

**Keywords:** breast cancer, patient navigation, systems of care, breast cancer diagnosis, wait time, breast cancer surgery

## Abstract

Introduction

Diagnostic delays for breast problems is a current concern in British Columbia and diagnostic pathways for breast cancer are currently under review. Breast centres have been introduced in Europe and reported to facilitate diagnosis and treatment. Guidelines for breast centers are outlined by the European Society for Mastology (EUSOMA). A Rapid Access Breast Clinic (RABC) was developed at our hospital applying the concept of triple evaluation for all patients and navigation between clinicians and radiologists. We hypothesize that the Rapid Access Breast Clinic will decrease wait times to diagnosis and minimize duplication of services compared to usual care.

Methods

A retrospective review was undertaken looking at diagnostic wait times and the number of diagnostic centres involved for consecutive patients seen by breast surgeons with diagnostic workups performed either in the traditional system (TS) or the RABC. Only patients presenting with a new breast problem were included in the study.

Results

Patients seen at the RABC had a decreased time to surgical consultation (33 vs 86 days, p<0.0001) for both malignant (36 vs 59 days, p=0.0007) and benign diagnoses (31 vs 95 days, p<0.0001). Furthermore, 13% of the patients referred to the surgeon in the TS without a diagnosis were eventually diagnosed with a malignancy and waited a mean of 84 days for initial surgical assessment. Of the patients seen at the RABC, 5% required investigation at more than one institution compared to 39% patients seen in the TS (p<0.0001). Cancer patients had a shorter time from presentation to surgery in the RABC (64 vs 92 days, p=0.009).

Conclusion

The establishment of the RABC has significantly reduced the time to surgical consultation, time to breast cancer surgery, and duplication of investigations for patients with benign and malignant breast complaints. It is feasible to introduce a EUSOMA-based breast clinic in the Canadian Health Care System and improvements in diagnostic wait times are seen. We recommend the expansion of coordinated care to other sites.

## Introduction

Wait times for the diagnosis of benign and malignant breast problems is a current concern in Canada and in British Columbia. Reports have documented increasing wait times for breast cancer diagnosis and treatment in Canada [1-4] and our aging population is putting a further strain on our publically funded health care system. Patients with benign presentations and less suspicious imaging findings tend to wait longer for a diagnosis in a publically funded health care system, but it is well documented that these patients have similar anxiety to those patients with malignant findings [5-6]. Furthermore, a proportion of patients with less suspicious presentations will be diagnosed with breast cancer [7].

In British Columbia (BC) it has been recognized that patients can become confused and overwhelmed as they progress through multiple community and hospital diagnostic imaging centers in order to diagnose their breast problem [8-9]. Although streamlined diagnostic pathways have been recommended to improve wait times [9] and patient experiences, they are not currently implemented in British Columbia. The Screening Mammography Program of BC has implemented a fast track booking policy for abnormal screening mammograms and many breast radiologists are fast tracking breast investigations; however, these approaches are not consistently available and many patients continue to have prolonged wait times to diagnosis of benign and malignant breast conditions in BC [10].

Breast centres have been developed in Europe to facilitate diagnosis and treatment of breast problems [11-12]. Berry [13] demonstrated a high level of patient satisfaction with a one-stop diagnostic clinic. Guidelines for breast centers in the United States and Europe recommend patient navigation with breast care nurses that facilitate next steps in the diagnostic and treatment process [11-12, 14]. Studies of patient navigation demonstrate an improvement in compliance with quality indicators [15-16] as well as an improvement in the timeliness of breast cancer care [17]. Other Canadian provinces have implemented similar strategies [18-21] and have shown improved wait times compared to BC [10].

A Rapid Access Breast Clinic (RABC) was established in 2009 at our center following the guidelines for breast centers outlined by the European Society of Mastology (EUSOMA) [12]. The program was implemented using Lower Mainland Innovation and Integration Funding, a pay for performance funding model established by the provincial government. The clinic provided triple evaluation of patients with close collaboration between clinicians and radiologists, facilitated by clinical pathways and nurse navigation. The development of the RABC in conjunction with the radiology department at our center created a unique situation in which the breast surgeons saw patients managed by two separate diagnostic pathways.

When the RABC was opened in 2009, it was planned to be the initial step towards coordinated care throughout our province. However, the Pay for Performance funding model was discontinued by the government [22] resulting in a clinic running with reduced staffing, and referral pathways in our area were blended. With changes in funding, different referral and staffing models have been tried and wait times monitored, but the most effective results were seen between 2009-2012. Wait times for diagnosis of benign and malignant breast conditions continue to be a concern in BC and at our center have returned to wait times seen before the implementation of the RABC. We continue to look for improvements in our care and to do this we have reviewed the effect of coordinated care in the RABC when the EUSOMA-based model was functioning.

We hypothesize that the RABC approach with coordinated care will decrease wait times to diagnosis and decrease duplication of services compared to standard care.

## Materials and methods

A retrospective review of a prospectively maintained breast clinic data base was undertaken to look at diagnostic wait times, treatment times, and the number of preoperative diagnostic centres involved for consecutive patients seen by the three breast surgeons in November and December 2009. This time frame was chosen as it was six months following the implementation of the clinic, allowing for stabilization of referral pathways but prior to changes in the Screening Mammography program implemented in 2010. A chart review of patients seen in the surgeon’s private offices during the same time frame was undertaken to obtain the corresponding information from patient charts. Approval for the study was obtained from the University of British Columbia Providence Health Care Review Board.

The RABC was established to offer a single site for coordinated clinical and radiological assessment of breast problems. Patients were referred to the clinic with either an abnormal screening mammogram through the screening mammography program at our hospital or were referred to the clinic by their family physician (FP) for assessment of a breast symptom. In the first year (2009-10) 1582 patients were assessed at the RABC with 472 referred through the screening program and 1110 referred with breast symptoms from the FPs. Eighty-five percent of patients were given their diagnosis within 21 calendar days of referral to the RABC—the target set for the Pay for Performance Funding. Most diagnostic workups that were delayed beyond 21 days were related to patient scheduling preferences or the need to schedule investigations such as stereotactic core biopsy or magnetic resonance imaging (MRI) at additional sites.

**Figure 1:**
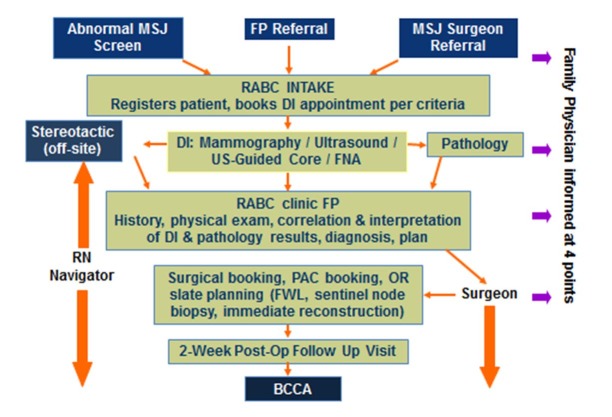
Care Pathways in the Rapid Access Breast Clinic MSJ= Mt St Joseph Hospital, FP= Family Physician, RABC= Rapid Access Breast Clinic, BCCA= British Columbia Cancer Agency, DI=Diagnostic Imaging, US=Ultrasound, FNA= Fine Needle Aspiration, OR=Operating Room, PAC= Preadmission Clinic, FWL=Fine Wire Localization, RN=Registered Nurse.

In our region, most breast diagnostic services offer mammography, ultrasound, and ultrasound-guided core biopsy. MRI, stereotactic core biopsy, and fine wire localization have been planned at different hospitals in our city as no one hospital had the capacity to manage the necessary volume of procedures. Our hospital offers on-site mammography, breast ultrasound, ultrasound-guided biopsy and mammographic and ultrasound-guided fine wire localization. It is the regional high volume breast surgery site performing 17-20% of general surgical breast procedures in the province over the past 10 years. Patients requiring stereotactic core biopsy, MRI or MRI-guided biopsy have those investigations coordinated by the RABC at regional imaging sites offering those investigations. When FPs refer to the program, they agree to allow the program to arrange and manage all subsequent care related to the presenting breast complaint or symptom. All appointments, investigations, and assessments are coordinated by the clerical staff following clinical pathways overseen by the radiologists and surgeons. The nurse navigator speaks with all patients that are recommended by radiology to have a core biopsy. After all investigations are completed, the patient is seen and examined in our clinic by the clinic FP who then correlates the physical examination, investigations, and biopsy results and either discharges back to the community for ongoing care or refers the patient to a breast surgeon as appropriate. If a patient is referred to the surgeon, the nurse navigator coordinates appointments and diagnostic and surgical bookings. The care provided in the clinic is shown in Figure 1. Surgeons working in the program met the criteria for high volume breast surgeons as outlined by EUSOMA [12]. Nurse navigators and FPs in the RABC were hired based on significant clinical experience with cancer patients. They then received breast-specific training from the breast surgeons beginning with training materials from the general surgery resident breast curriculum followed by clinical orientation, clinical encounters supervised by the breast surgeons, and initial RABC shifts scheduled to coincide with the presence of the breast surgeons in the RABC.

With the introduction of the RABC the breast surgeons working at our regional center saw patients in one of two locations- either patients that had been seen through the RABC or patients referred to their private office that would have received standard care (hereafter referred to as the Traditional System, or TS). This latter group of patients would have had investigations at 12 other imaging centers and were referred for surgical assessment and treatment of breast complaints or breast cancer.

In the TS, each imaging investigation requires a requisition. Patients with a breast complaint are initially seen by their FP, who then refers them to a radiology diagnostic centre for the appropriate test. The radiology report goes back to the FP who discusses the results with the patient and then arranges the next steps in the diagnostic workup. When a patient attends a different diagnostic imaging center, mammograms and/or breast ultrasound are often repeated. The patient is referred to a surgeon as needed. Patients referred to the surgeon’s private office were triaged by the surgeon on receiving the referral, giving urgent appointments to patients with cancer or high risk lesions and next available appointments to patients referred with not yet diagnosed or benign conditions.

Once seen by the surgeon, all patients were placed on the same surgical wait list for breast surgery at our regional center, regardless of the location where the consultation was performed (RABC or private office). The date they were placed on the surgical wait list was the date surgery was recommended.

The primary endpoint of the study was time from presentation to surgical consultation. This time was chosen as this was the common point in both diagnostic pathways, and patients were combined on a surgical wait list once they had been assessed by the surgeon. The secondary endpoint was the number of diagnostic radiology centers that patients needed to visit in order to obtain their diagnosis. Patients seen in TS (standard care) were compared to patients seen at the RABC. Only patients presenting with a new breast problem were included in the study. Patients that had previously been assessed by the breast surgeon, follow-up patients, patients presenting for a second opinion, and patients with chronic breast conditions were excluded. Because of some of the logistical issues that were encountered in setting up the new referral system, two patients that should have been seen in the RABC by the surgeon were seen in the private office. Both of these patients had presented with abnormal screening mammograms at our center and had diagnostic workups partially organized by their own FPs in the TS. The analysis was done on an intention-to-treat basis and those two patients were included in the RABC analysis group.

Statistical analysis was performed using a Student’s t-test for continuous variables, and a chi-square test was performed for categorical variables. A p-value of <0.05 was considered significant.

## Results

During the study period, 373 patients were seen by the three breast surgeons for consultation. There were 240 patients seen with a new breast problem, 64 in the RABC and 178 in the office. During the same time period, 192 new patients were assessed by the clinic FP in the RABC with 85% of the patients receiving their diagnosis within 21 calendar days. In the study group, 99% of patients were female with one male seen in the TS and two males seen in the RABC. In the RABC the surgeons saw 24 (38%) patients with a known cancer diagnosis and 40 (62%) patients with a benign or indeterminate diagnosis. In the office, 44 (25%) patients were referred with a known cancer diagnosis and 134 (75%) patients were referred with a benign diagnosis, indeterminate diagnosis, or an incomplete diagnostic workup. The referral diagnoses for both groups of patients is outlined in Table 1. On reviewing the charts of the TS patients with a benign diagnosis, 65 of 134 (49%) patients saw the surgeon to complete a diagnostic workup and would not have been referred to the surgeon in the RABC. The tumor characteristics, treatment, and follow-up of patients presenting with breast cancer are outlined in Table 2. Two RABC cancer patients declined treatment, and there were two patients in the RABC and one in the TS that we did not have follow-up or treatment information for.

**Table 1:** Clinical Presentation of Patients Seen by the Surgeon in the Traditional System (TS) and at the Rapid Access Breast Clinic (RABC) and Included in the Study NYD=Not Yet Diagnosed, DCIS=Ductal Carcinoma in Situ.

Referral Reason	TS (n=178 )	RABC (n=64 )
Invasive Cancer	31 (18%)	22 (34.5%)
DCIS	13 (7%)	2 (3%)
High risk lesion on core biopsy	16 (9%)	9 (14%)
Abnormal Screening mammogram NYD	31 (18%)	0
Mass	61 (34%)	15 (23.5%)
Cyst	6 (3%)	0
Nipple discharge	7 (4%)	6 (9%)
Breast pain	7 (4%)	0
Breast/nipple change	4 (2%)	5 (8%)
Breast Abscess	0	2 (3%)
other	2 (1%) gynecomastia, risk assessment	3 (5%) gynecomastia-2 foreign body
Upgrade to cancer diagnosis after seeing surgeon	17 (13%)	0

**Table 2:** Tumor Characteristics, Treatment, and Outcomes for Patients with Breast Cancer TS=Traditional System, RABC= Rapid Access Breast Clinic, DCIS=Ductal Carcinoma in Situ, ER+=Estrogen Receptor positive, Her2+=Her2/neu protein positive.

		TS	RABC
Invasive Cancer (%)		31 (72%)	18 (90%)
DCIS (%)		12 (28%)	2 (10%)
Average tumor size (mm)		20.1	20.4
Tumor grade (%)	grade 1	6 (17%)	2 (11%)
	grade 2	19 (53%)	9 (47%)
	grade 3	11 (31%)	8 (42%)
Total mastectomy (%)		17 (40%)	11 (55%)
Axillary dissection (%)		9 (21%)	9 (45%)
ER+ (%)		29 (67%)	12 (60%)
Her2+ (%)		4 (9%)	1 (5%)
Malignant nodes (%)		10 (23%)	8 (40%)
Chemotherapy (%)		9 (21%)	7 (35%)
Radiation therapy (%)		29 (67%)	11 (55%)
Hormone therapy (%)		22 (51%)	11 (55%)
Mean follow-up (months)		63.6	68.8
Breast cancer recurrence (%)		6 (14%)	3 (15%)
Breast cancer death (%)		3 (7)%	2 (10%)

**Figure 2:**
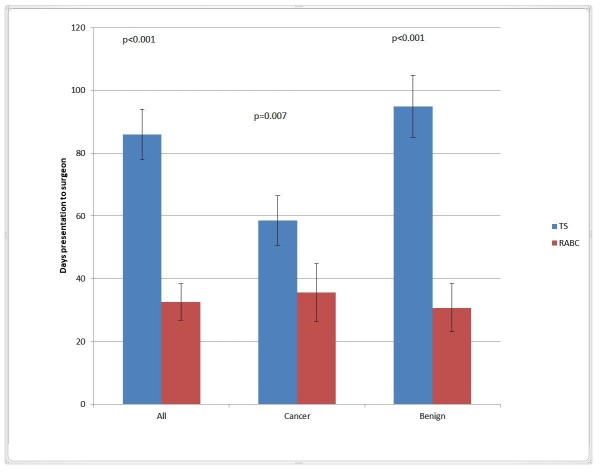
Time from Presentation to Surgical Assessment TS=Traditional System, RABC=Rapid Access Breast Clinic, Bars represent mean time in days and whiskers represent 95% Confidence Interval.

Patients seen at the RABC had a decreased time to surgical consultation (mean of 33 vs 86 days, p<0.0001) for malignant (36 vs 59 days, p=0.0007) and benign diagnoses (31 vs 95 days, p<0.0001). The time from presentation (finding a lump or an abnormal screening mammogram) to seeing the surgeon is outlined in Figure 2. Furthermore, 17 (13%) of the patients referred with a benign or indeterminate diagnosis in the TS were diagnosed with breast cancer after seeing the surgeon. They waited a mean of 84.2 days to see the surgeon and longer for diagnosis, as they were not diagnosed at the time of surgical consultation.

For the patients presenting with breast cancer and having initial treatment with surgery, the time from surgical consultation to surgery was 33 days in the TS and 31 days in the RABC (p=0.78). However, the time from presentation to surgery for cancer patients was decreased for patients managed through the RABC (64 vs 92 days, p=0.009). For cancer patients that had a core biopsy prior to seeing the surgeon, the time from presentation to core biopsy was shorter in the RABC (18 vs 38 days, p=0.002) as was the time from core biopsy to seeing the surgeon (15 vs 25 days, p=0.01).

In the RABC, 61 (95%) patients had their diagnosis at one center and three (5%) patients had diagnostic studies at other centers. In contrast, in the TS only 61% attended a single diagnostic center with 39% attending two or more diagnostic centers (p<0.0001). The time patients waited stratified by the number of diagnostic centers they visited is outlined in Table 3. A patient attending four diagnostic centers had a mammogram at one center, an ultrasound at a different center, an ultrasound-guided biopsy at a third center, and a stereotactic core biopsy at a fourth center. Of the patients having only mammograms, ultrasound, and ultrasound-guided biopsy in the TS, 31.5% of the patients attended more than one diagnostic imaging center and were more likely to have attended one diagnostic center for these tests in the RABC (p<0.0001). Patients referred with a breast cancer diagnosis were more likely to have been seen in a single diagnostic center for mammogram, ultrasound, and ultrasound-guided core biopsy when seen at the RABC than in the TS (p=0.021).

**Table 3:** Time to See Surgeon by Number of Diagnostic Centers Attended TS=Traditional System, RABC=Rapid Access Breast Clinic.

Number of Diagnostic Centers attended	Number of Patients	Mean time to see surgeon (days)
RABC 1	61	33
RABC 2	3	30
TS 1	109	79
TS 2	59	80
TS 3	10	116
TS 4	1	166

Diagnostic wait times for patients presenting with screen detected abnormalities and breast symptoms are compared in Table 4. Seventy-two patients were seen in the TS after presenting with an abnormal screening mammogram and waited a mean of 82 days to see the surgeon. Patients with presentations other than an abnormal screen were classified as symptomatic and waited a mean of 92 days to see the surgeon in the TS.

**Table 4:** Wait Time for Screening and Symptomatic Patients TS=Traditional System, RABC=Rapid Access Breast Clinic. *includes a patient who had been seen in consultation at the surgeon's private office but screening done at MSJ, analyzed on intention-to-treat basis in RABC group.

	RABC Time to see surgeon (days)	TS Time to see surgeon (days)
Patients abnormal screen no stereotactic biopsy	40 (n=2*)	69 (n=42)
Patients abnormal screen with stereotactic core biopsy	70 (n=2*)	99 (n=30)
Patients with breast symptoms	31 (n=60)	92 (n=102)

## Discussion

This data confirms that wait times to diagnosis of breast problems in our area are exceeding recommended targets for patients receiving usual care. The RABC was set up in 2009 as what was planned to be the initial step in developing coordinated diagnostic care for benign and malignant breast problems in our area. The Innovation Funding model that started the clinic was not continued after 2012 [22], and thereafter patients seen through the RABC entered the clinic at different points in their diagnostic journey. Different models of care have been tried and wait times for diagnosis and surgery have increased again in our area similar to findings from before the introduction of the RABC. This has prompted us to re-evaluate our experience from when the RABC was implemented using a EUSOMA-based model in 2009. Because patients were most clearly cared for in two separate diagnostic pathways, these results will reflect the effect of coordinated care compared to usual care. The unique situation that arose with surgeons working simultaneously in two separate diagnostic systems with the development of the RABC gave us an opportunity to study the effect of coordinated and navigated care in a public health care system.

The effect of wait times in cancer treatment is controversial. Some authors have found delays to treatment to have a negative effect on survival, but this has not been demonstrated in all studies [23-24]. Studies of patients in the screening mammography programs in Canada have demonstrated that patients with screen-detected lesions had a 25-44% improvement in survival as a result of screening [25-26], attributed to screening identifying lesions at an earlier stage. Coldman’s [25-26] studies do not inform us as to time frames in which the benefit of early detection applies nor relate to diagnostic wait times, but such data suggests that it is appropriate to optimize our breast care system to minimize diagnostic delays.

The results from the RABC show that a breast unit located in a hospital offering readily available diagnostic techniques (mammogram, ultrasound, and ultrasound-guided biopsy) was able to offer most patients diagnostic workups in the 21-day time frame recommended by EUSOMA [12]. It should therefore be feasible to expand this model to most centers, and what would be required is the infrastructure for navigation of patients. Like at our center, patients requiring stereocore biopsy and MRI can have their care coordinated with the regional centers offering that service. Decreasing duplication of studies should increase capacity in the system.

There are currently no Canadian recommendations for wait times for patients presenting with breast symptoms such as a mass or nipple discharge and very little is known about these patients as diagnostic wait times information is gathered from provincial screening programs. However, the mean wait time for these patients to see a surgeon from presentation is 92 days compared to 82 days for patient presenting from screening, likely reflecting the additional time that it would take to see their family physician and arrange the first diagnostic imaging test. Even allowing for time from presentation to imaging, and imaging to surgeon, these wait times are far exceeding Canadian recommendations for image-detected lesions: five weeks from abnormal mammogram (no biopsy) and seven weeks from abnormal mammogram (with biopsy) [10]. These findings suggest that our standard care wait times in BC are longer than those reported in other areas in Canada for breast cancer diagnosis [4, 27] but this can only be inferred as time frames measured varied between different studies. Nashed [27] found that patients that had a longer travel time had a shorter wait time for diagnostic testing in Manitoba. When our data is compared to Baliski’s [4] data (from a smaller BC community) a similar pattern is suggested.

Many centers now monitor the time from core biopsy to surgery. We did not use the time from core biopsy to surgery for this study as the core biopsies were often arranged by the surgeon’s office in the TS (either for before or after the patient consultation date) and was therefore not felt to reflect the diagnostic process that was the purpose of the RABC and this study. Similarly, because the patients were placed on a single, combined surgical wait list, the time to surgery was not felt to reflect the differences of the two diagnostic systems, yet the improvement in diagnostic time for cancer patients in the RABC results in a significant decrease in time from presentation to surgery in the RABC compared to the TS. Because the common event between the two diagnostic systems was seeing the surgeon and because the patients were then combined on a surgical wait list, the date of surgical consultation was chosen as the study end point. Nashed, et al. [27] report that 21% of patients had a surgical consultation prior to core biopsy diagnosis of breast cancer in Manitoba during the same time frame, suggesting that similar diagnostic processes are occurring in other Canadian provinces.

The purpose of this study was to look at the effect of a EUSOMA-based model in improving wait times for breast cancer diagnosis, and it has demonstrated significant delays in breast diagnostics and movement of many patients between different diagnostic centers in our area. Prior to the study, based on experience and previous work [8-9], the authors hypothesized that the movement between different diagnostic centers and the multiple handovers in care between radiology and clinicians would result in diagnostic delay; the findings of delay to seeing the surgeon, longer wait times between presentation and core biopsy and core biopsy to surgical consultation, and 39% of patients attending multiple diagnostic centers supports this theory. Detailed data about wait times for each step in the diagnostic process was not available in the surgeons records as this information came from care prior to involvement of the surgeon; this is a limitation of the study. Further work is planned to explore the details of the delays to help inform improvements, but the significant decrease in wait times shown with the coordinated care in this study demonstrates that coordination of radiologic and clinical care addresses the pitfalls in the usual care pathways.

There have been concerns about the cost of coordinated care. In a randomized controlled trial Wagner [28] showed that nurse navigator support for patients early in their course improved patient experience, decreased problems, and had similar costs for breast cancer patients compared to usual care. Donaldson [29] found that patient navigation improved the number of patients diagnosed within recommended time frames and was cost effective. We found that a significantly greater number of patients in the TS had attended more than one diagnostic imaging center. When we exclude patients having stereotactic core biopsy, which would usually require attending additional centers, in our area, we still found that 31.5% of patients attended more than one diagnostic imaging center for their diagnosis. This means that there would be an additional testing cost for repeat examinations, transport of films, and rereading of films for these patients, representing a significant cost to the health care system. In addition, in our TS the FP arranges each step of the diagnostic workup and those costs were saved in the RABC model. This study did not specifically assess the costs of the RABC care; however, in order to continue funding the RABC, the Health Services Purchasing Organization/Ministry of Health requested a costing study of the RABC in 2011. This data demonstrated cost savings over usual care [30].

One of the limitations of this study is the retrospective nature of the data that was obtained. Although data was prospectively collected for patients seen in the RABC, prospective data is not collected in the surgeon’s private offices, so a retrospective design was necessary to compare wait times with the two diagnostic processes. Another limitation was determining the presentation date. For the patients seen in the RABC, the date of referral from the FP is recorded and this was used as the start date for the problem. In the surgeon’s office, the date of referral does not correspond to the beginning of the diagnostic workup as the patients have much of the diagnostic workup by the family physician. The surgeon’s recording of when the problem began was used as the start date, but some patients may have had the problem for some time before seeing the FP and for these patients the time from start to seeing the surgeon may seem longer due to this factor. However, many patients seeing the surgeon had their diagnostic workup continued by the surgeon, so for this group of patients the time from start to seeing the surgeon would have underestimated the diagnostic workup time. Putting these two factors together the time from the breast problem starting to seeing the surgeon was felt to be the best possible estimate of the time in the diagnostic workup time in the TS.

An important finding in our study was that 17% of patients seeing the surgeon referred with a benign diagnosis or incomplete diagnostic workup in the TS were diagnosed with cancer after seeing the surgeon, yet waited a mean of 84 days to see the surgeon for assessment. While this could also occur in the RABC model, (for example, a diagnosis after excisional biopsy of a high risk lesion) the patient would have seen the surgeon almost two months sooner in the RABC due to the coordinated care. This is due to the coordination of diagnostic tests, but also utilization of family physicians in the RABC, which keeps the wait times to surgical consultation down by having patients with benign diagnoses see the FP. The coordination of investigations decreases duplicates and should increase capacity within radiology. The systematic involvement of FPs in our clinic increases capacity for surgical consultation in a timely manner for those patients requiring surgical intervention and treatment. Coordination of the radiological and clinical care decreases the number of episodes of hand over of care, improves wait times, and achieves wait times results that comply with the targets recommended by EUSOMA.

## Conclusions

Wait times for breast cancer diagnosis are exceeding recommended targets for patients receiving usual care in our area, and implementation of a EUSOMA-based clinic with coordinated care is able to reduce wait times to meet national and international benchmarks. Furthermore, patients presenting with less suspicion for malignancy are also diagnosed in a timely fashion. Single center imaging diagnosis with ultrasound-guided biopsy and coordination of care reduces time to diagnosis but will also reduce the number of duplicate studies being performed and the number of additional visits or calls to family physicians to arrange next steps. The EUSOMA model of care can be effectively implemented in the Canadian health care system. We recommend enhancing coordination of care again at our center and the expansion of coordinated care to other centers.
